# Reactivation of Vogt-Koyanagi-Harada disease under control for more than 6 years, following anti-SARS-CoV-2 vaccination

**DOI:** 10.1186/s12348-021-00251-5

**Published:** 2021-07-05

**Authors:** Ioannis Papasavvas, Carl P. Herbort

**Affiliations:** Retinal and Inflammatory Eye Diseases, Centre for Ophthalmic Specialized Care (COS), Teaching Centre Clinic Montchoisi, Lausanne, Switzerland

**Keywords:** Vogt-Koyanagi-Harada disease: Indocyanine green angiography, Autoimmune stromal choroiditis, Anti-SARS-CoV-2 vaccination

## Abstract

**Background/purpose:**

Vogt-Koyanagi-Harada (VKH) disease is a primary stromal choroiditis with bilateral granulomatous panuveitis. If initial-onset VKH is treated early and relentlessly the disease can be controlled and even “cured” in a substantial number of cases. We are reporting on a patient treated early and in a sustained fashion who was inflammation free for seven years but who presented a reactivation 6 weeks after the second dose of anti-SARS-CoV-2 vaccination.

**Case report:**

A 43-year-old woman presented with severe initial-onset VKH disease which was brought under control using steroidal and non-steroidal Immunosuppression (mycophenolic acid and cyclosporine) with additional infliximab infusions because of the persistence of subclinical choroiditis identified on ICGA. Under infliximab alone disease had been inflammation free with no subclinical disease and absence of sunset glow fundus for 6 years. However, following anti-SARS-CoV-2 vaccination, severe resurgence of the disease occurred with exudative retinal detachments. Disease was rapidly brought again under control with oral prednisone (1 mg/kg) therapy and a new loading scheme of infliximab therapy.

**Conclusion:**

VKH disease results from an autoimmune process directed against melanocyte associated antigens which can be controlled when early and sustained immunosuppressive treatment is introduced. It seems that anti-SARS-CoV-2 vaccination can be at the origin of reactivation of long-time controlled disease.

## Introduction

Vogt-Koyanagi-Harada (VKH) disease is a primary stromal choroiditis [1, 2] caused by an autoimmune reaction against melanocyte associated proteins [3, 4]. Eye involvement is associated with systemic manifestations including inflammation of the meninges (CSF mononuclear pleiocytosis) and auditory disturbances [5, 6].

Since the mid-1990s, precise investigation of choroidal inflammation is obtained thanks to ICGA, allowing to detect subclinical disease during initial onset disease and to monitor occult subclinical recurrence during follow-up [7–9]. To a lesser extent this is also possible with Enhanced Depth Imaging Optical Coherence Tomography (EDI-OCT) [10].

We report a case of severe VKH disease for whom inflammation was brought under control after several months with combined steroidal and non-steroidal immunosuppression and who was inflammation free with a maintenance treatment of infliximab every 10 weeks for 6 years but presented a severe reactivation 6 weeks after the second dose of Pfizer anti-SARS-CoV-2 vaccination.

## Case report

**Fig. 1:**
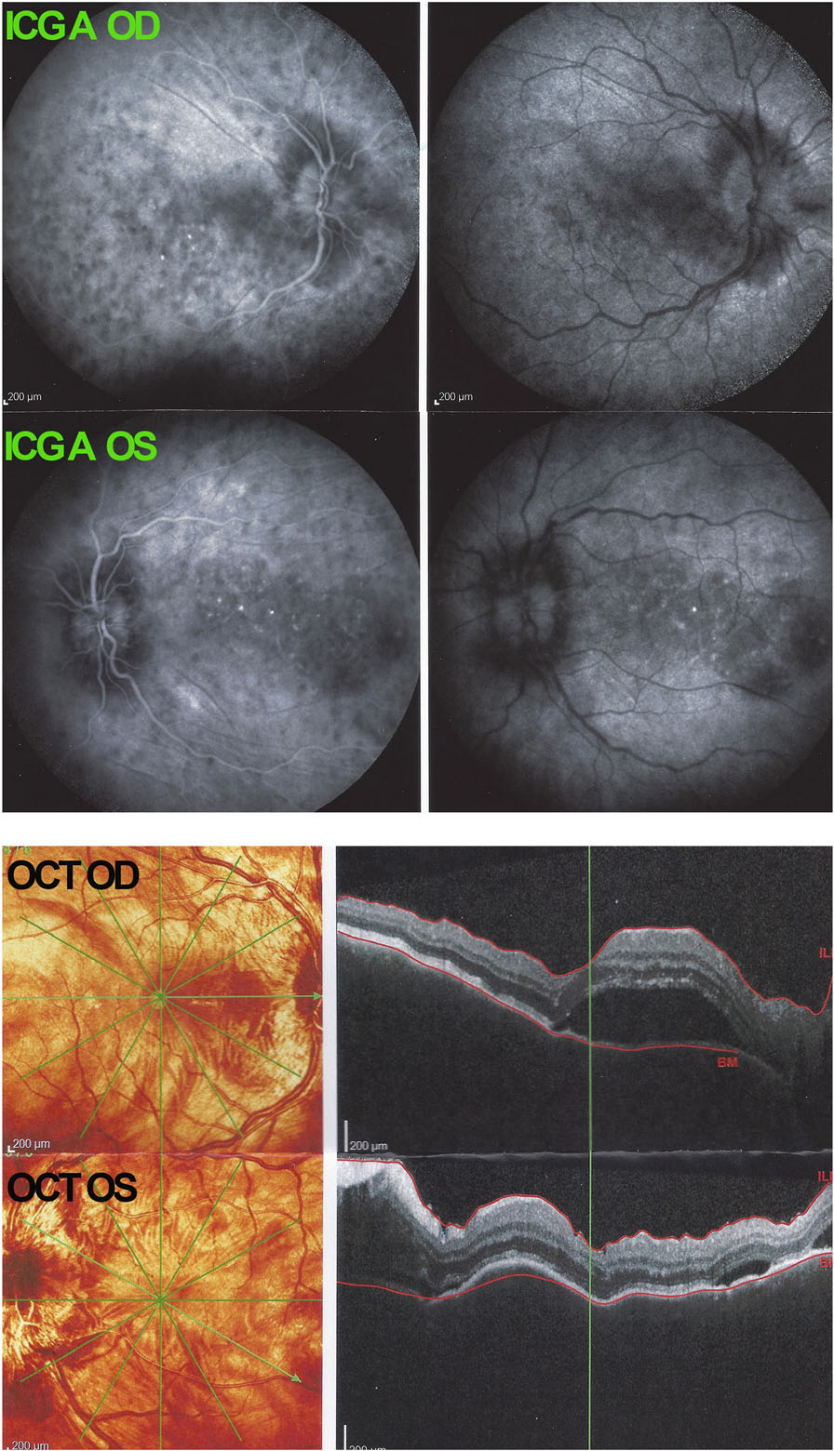
Case of VKH at presentation. ICGA picture ODS shows exudative detachments and numerous HDDs. OCT shows retinal folds and subretinal fluid and very thickened choroids impossible to measure on EDI-OCT

**Fig. 2:**
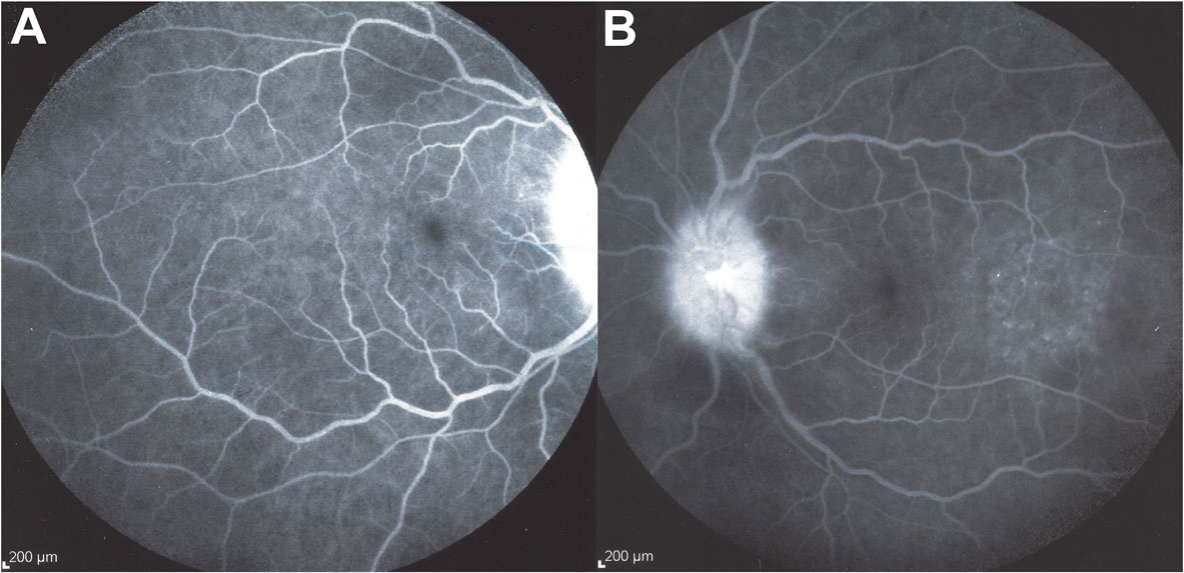
FA: exsudative retinal detachements around optic disc (**a**) and temporal to the fovea OS (**b**)

A 43-year-old woman consulted the Centre for Ophthalmic Specialised Care (COS) in Lausanne, Switzerland, because of bilateral decreased visual acuity, photophobia, and eye tenderness. One month earlier she had started to experience headaches, increased scalp sensitivity, neck stiffness and a red eyes treated with drops containing an antibiotic and dexamethasone for two weeks. We saw the patient 5 weeks after the prodromal symptoms. Best corrected visual acuity (BCVA) was reduced to 0.7 (OD) and 0.8 (OS) (Snellen chart). Intraocular pressure (IOP) was 9 mmHg ODS. Significant non-granulomatous anterior chamber inflammation was noted with laser flare photometry (LFP) values of 137.5 ph/ms OD and 148.2 ph/ms OS (normal values 4–6 ph/ms) and there were 1+ (OD) and 2+ (OS) cells in the vitreous. Optical coherence tomography showed retinal folds and subretinal fluid and enhanced depth imaging OCT (EDI-OCT) showed such an increased choroidal thickness that no measurement was possible (Fig. 1). Fluorescein Angiography (FA) (Fig. 2) showed exudative retinal detachments which were also seen on Indocyanine angiography (ICGA) that also showed numerous hypofluorescent dark dots (HDDs) (Figs. 1 and 2).

**Fig. 3:**
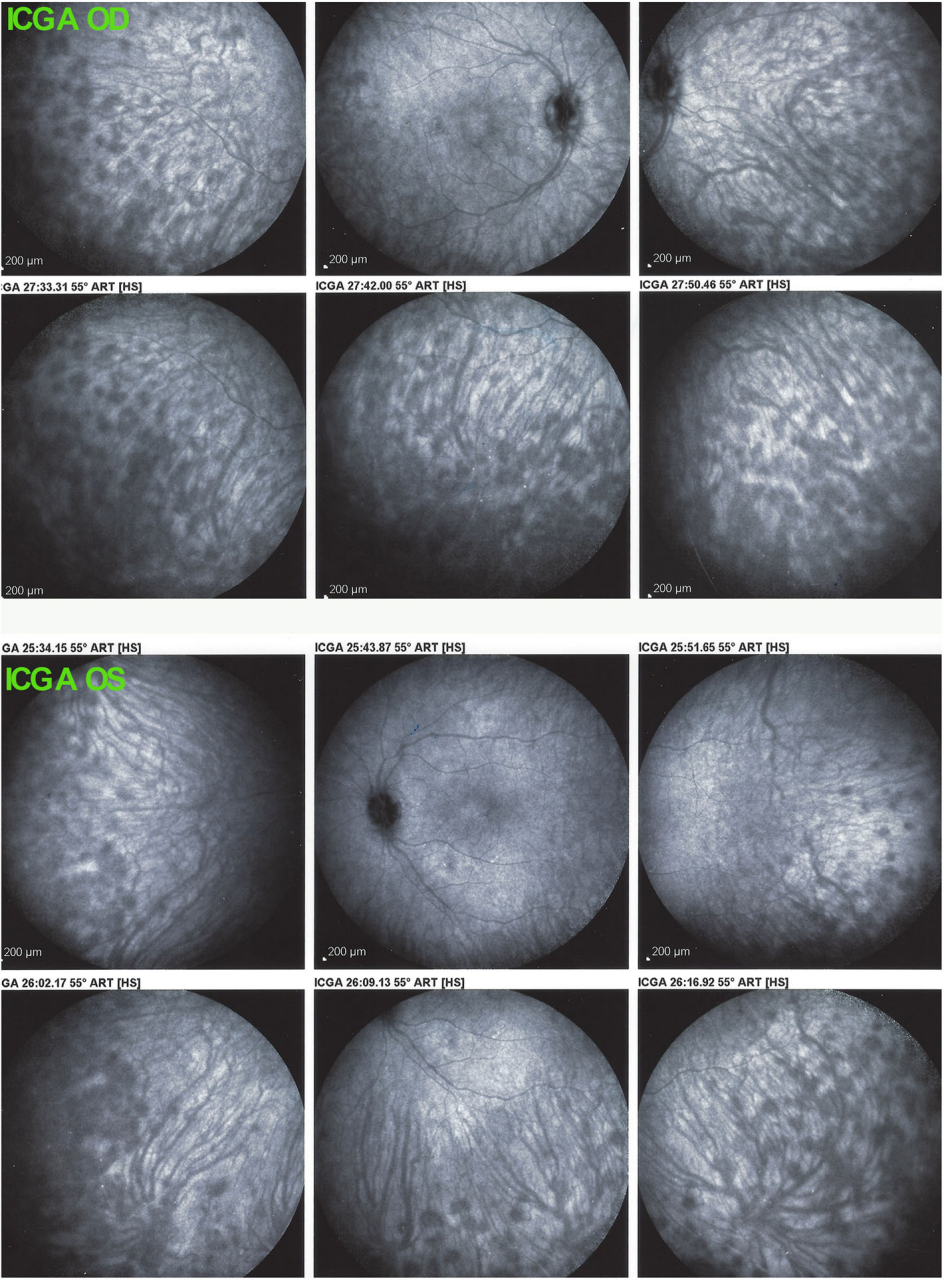
ICGA ODS of VKH patient at the time of prednisone discontinuation. ICGA shows re-apparition of HDDs at the moment of prednisone discontinuation, despite mycophenolic acid and cyclosporine therapy that led to the introduction of infliximab treatment

Because this initial-onset VKH disease was beyond the 3–4-week interval considered to be within the therapeutic window of opportunity for cure of the disease [11], we decided to apply vigorous and sustained treatment inflammation suppressive treatment. We started with intravenous methylprednisolone (1000 mg/day) for 3 days followed by oral prednisone with concomitant cyclosporine (4.5 mg/kg) and mycophenolic acid (Myfortic®, 720 mg twice daily). Upon tapering oral prednisone, subclinical choroiditis recurrence was noted on ICGA (Fig. 3) and infliximab (5 mg/kg) was added followed by resolution of all choroidal inflammation 3 months later.

**Fig. 4:**
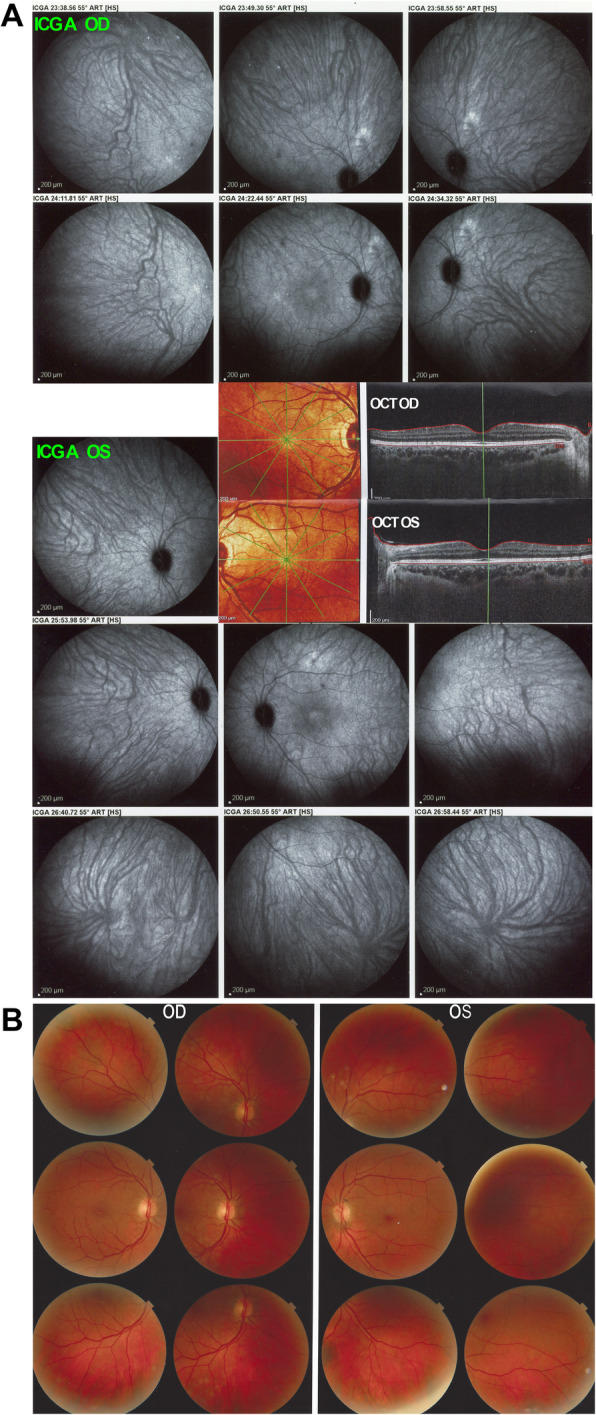
**a** ICGA and OCT after 6 years of infliximab treatment. ICGA pictures show a normal choroid devoid of HDDs and normal OCT images ODS (insert). **b** Fundus photographs ODS seven years after initial-onset VKH disease. Absence of notable sunset glow fundus 2 months prior to post-vaccination recurrence

Under this treatment ocular findings, including anterior chamber and vitreous inflammation, exudative retinal detachments and choroidal thickness reversed to normal 9 months after the start of disease. Except for infliximab, all other treatments were stopped, including oral prednisone within 9 months, cyclosporine within 13 months and mycophenolic acid within 22 months. Infliximab infusions were given every 6 weeks for 30 months and every 10 weeks during the 3 subsequent years, all disease parameters being under control with no disease activity, no occult choroiditis, and no sunset glow fundus (Figs. 4a & b).

**Fig. 5:**
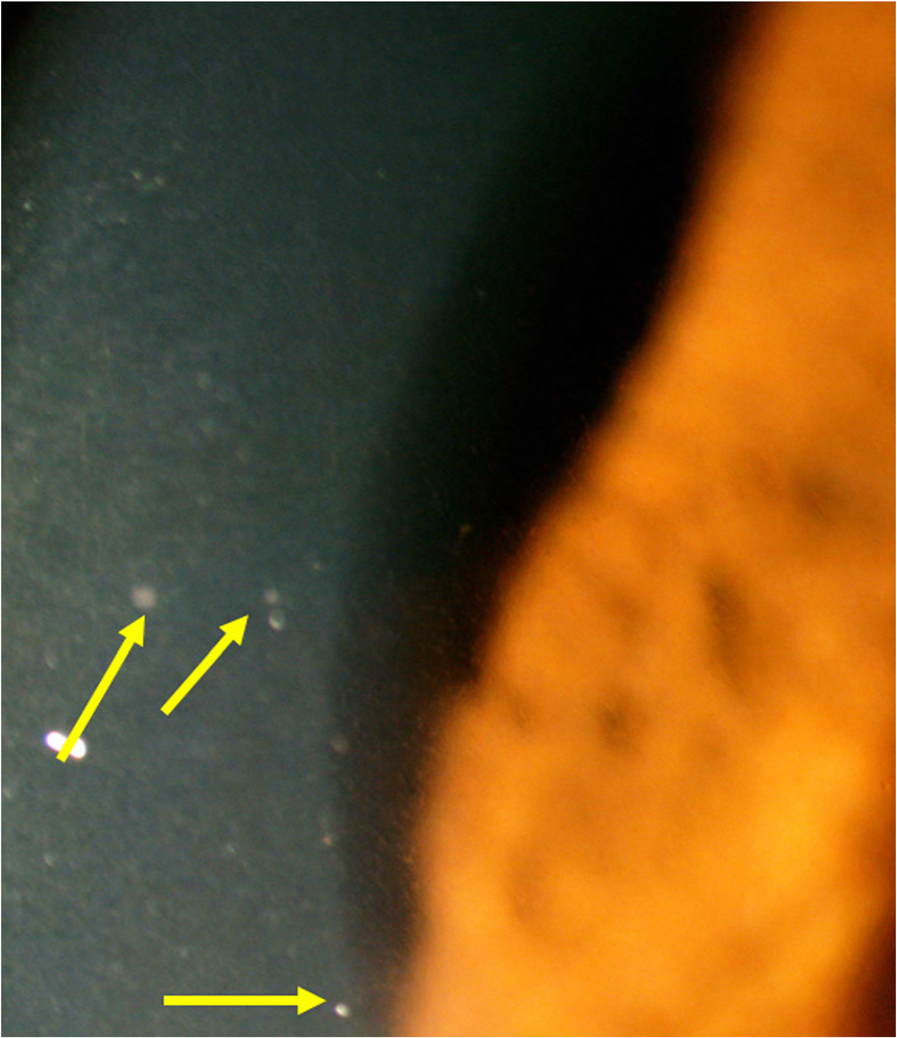
Recurrence of VKH disease after 6 inflammation free years. While the initial-onset disease was non-granulomatous, mutton-fat KPs were seen during post-vaccine recurrence OD (yellow arrows)

**Fig. 6:**
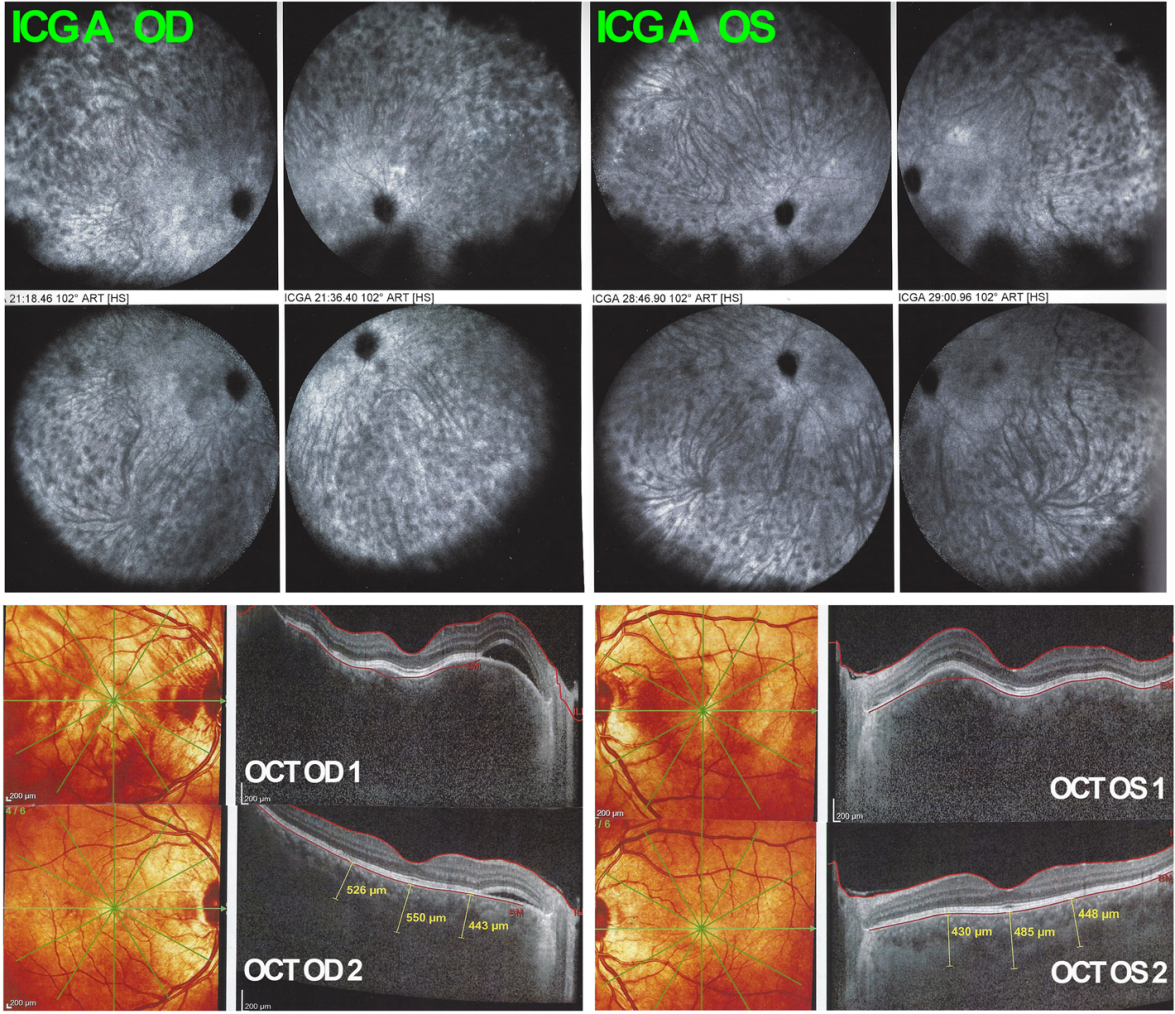
Recurrence of VKH after the second dose of anti-SARS-CoV-2 vaccination. On ICGA, numerous HDDs ODS dispersed over both fundi are present. OCT shows exudative retinal detachments and markedly thickened choroid not measurable OCT OD1 and OCT OS1. Thickness decreased substantially after 5 days of oral corticosteroids to 506 ± 50 μm OD and 454 ± 28 μm OG (OCT OD 2 & OCT OD 2)

However, 6 weeks after the second dose of the Pfizer anti-SARS-CoV-2 vaccine administration, the patient presented a severe reactivation of disease almost as pronounced as during initial-onset disease. Best corrected visual acuity (BCVA) remained full at 1.0 ODS. There was substantial anterior segment inflammation with laser flare photometry (LFP) values of 34.8 ph/ms OD and 64.2 ph/ms OS and this time, 3–4 small mutton-fat keratic precipitates OD were present **(**Fig. 5**)**. There were retinal folds and subretinal fluid on OCT and choroidal thickness was markedly increased and not measurable on EDI-OCT (Fig. 6). There were again numerous HDDs visible on ICGA **(**Fig. 6**).** After 5 days of oral prednisone (1 mg/kg), LFP anterior segment inflammation quasi normalised to 9.1 ph/ms OD and 7.0 ph/ms OS and choroidal thickness had again diminished substantially and increased thickness could again be measured with a mean of 506 ± 56 μm OD and 454 ± 28 μm (Fig. 6). The last infusion of infliximab having been administered 3½ weeks before the first vaccination and 7½ weeks before the second vaccination, this probably explains the fact that reactivation occurred only after the second vaccination. Infliximab was again administered following again a loading dose scheme with positive short-term evolution.

## Discussion

Many mild or moderate systemic side-effects after anti SARS-CoV-2 vaccination have been reported. There is probably a tendency, with the massive vaccination campaign similar to no other, to overattribute many adverse events to this vaccination. In our case, the delay of 6 weeks until reactivation of VKH inflammation seems fairly long to be linked to the vaccination. However, the fact that the disease was completely under control for 6 years and recured all of a sudden after the vaccination strongly points towards it being the trigger of this reactivation, taking into account that nothing in the health status, treatment or in the lifestyle of this patient had changed. Moreover, the reactivation of a complex immunological mechanism, probably needs some time to translate into disease resurgence.

It is interesting to note that, during the first 9 months after disease onset, when prednisone was tapered and stopped, the combination of cyclosporine and mycophenolic acid was unable to prevent occult reactivation of choroiditis that was detected by ICGA. This latter modality is known to be very sensitive, showing diligently that, for a given patient whether he/she responds to a particular therapy. Indeed, ICGA was shown to be very sensitive to rapidly detect whether a treatment was efficient or not and to fine-tune therapy in primary stromal choroiditis [12].

Another point of interest is that, thanks to sustained treatment no notable sunset glow fundus occurred [13]. VKH disease is classically a granulomatous disease. However initial-onset disease in this patient was non-granulomatous and granulomatous KPs developed only after the vaccination-triggered recurrence.

Numerous posterior inflammatory events after diverse vaccinations have been reported, including multiple evanescent dot syndrome (MEWDS) after influenza vaccination with at least 9 cases reported [14, 15], acute posterior placoid pigment epitheliopathy (APMPPE) after influenza vaccination [16] and unilateral acute idiopathic maculopathy (UIAM) after H1N1 influenza type A vaccination [17]. The inflammatory events in these cases occurred between 1 and 3 weeks after vaccination.

Three reports on development of VKH disease have been identified after Hepatitis B, yellow fever and BCG vaccinations .[18–20] These were all diseases that developed “de novo”.

A recent opinion paper on COVID-19 vaccination in immune-mediated diseases concluded that ocular (immune-mediated) side effects were very unlikely to happen, which should, however, not prevent us to be vigilant [21].

## Conclusion

Reactivation of VKH disease is another potential side effect of anti-SARS-CoV-2 vaccination to be added to the list of effects due to this vaccination. It should however be stressed that a substantial number of side-effects have to be expected in such an unprecedented large-scale vaccination. Most of these side-effects are benign and, in case of more severe side-effects, like for our patient, treatment is usually available. It is quite obvious that, as far as mRNA vaccines are concerned, and probably also other anti-SARS-CoV-2 vaccines, the benefit of vaccination largely outweighs the possible but very low risk of ocular side effects.

## Data Availability

For data, please refer to corresponding author.

## Abbreviations

VKH: Vogt-Koyanagi-Harada
CSF: Cerebro-spinal Fluid.
EDI-OCT: Enhanced Depth Imaging Optical Coherence Tomography
HDDs: Hypofluorescent dark dots
FA: Fluorescein Angiography
ICGA: Indocyanine Angiography
BCVA: Best corrected visual acuity
LFP: Laser flare photometry
